# Regio and stereoselective synthesis of anticancer spirooxindolopyrrolidine embedded piperidone heterocyclic hybrids derived from one-pot cascade protocol

**DOI:** 10.1186/s13065-018-0462-x

**Published:** 2018-09-01

**Authors:** Natarajan Arumugam, Abdulrahman I. Almansour, Raju Suresh Kumar, Dhaifallah M. Al-thamili, Govindasami Periyasami, V. S. Periasamy, Jegan Athinarayanan, Ali A. Alshatwi, S. M. Mahalingam, J. Carlos Menéndez

**Affiliations:** 1 Department of Chemistry, College of Science, King Saud University, P.O. Box 2455, Riyadh, 11451 Saudi Arabia; 2 Nanobiotecnology and Molecular Biology Research Laboratory, Department of Food Science and Nutrition, College of Food and Agricultural Sciences, King Saud University, Riyadh, Saudi Arabia; 3 Department of Atomic and Molecular Physics, MIT Campus, Manipal Academy of Higher Education, Manipal, Karnataka 576104 India; 4 Unidad de Química Orgánica y Farmacéutica, Departamento de Química en Ciencias Farmacéuticas, Facultad de Farmacia, Universidad Complutense, 28040 Madrid, Spain

**Keywords:** Spiropyrrolidine, Piperidone, Domino reactions, Chemo divergent multicomponent reactions, Antiproliferative activity, Apoptosis induction

## Abstract

**Background:**

Spiropyrrolidine tethered piperidone heterocyclic hybrids were synthesized with complete regio- and stereoselectively in excellent yield via *a* tandem three-component 1,3-dipolar cycloaddition and subsequent enamine reaction in [bmim]Br. The synthesized compounds were evaluated for their anticancer activity against FaDu hypopharyngeal tumor cells.

**Findings:**

Interestingly, most compounds displayed cytotoxicities similar to the standard anticancer agent bleomycin, with two of them (**5a** and **5g**) being slightly more active than the reference drug.

**Conclusion:**

Synthesized compounds have also been evaluated for their apoptosis-inducing properties in a cancer cell model, finding that treatment with compounds **5a**–**e** led to apoptotic cell death.
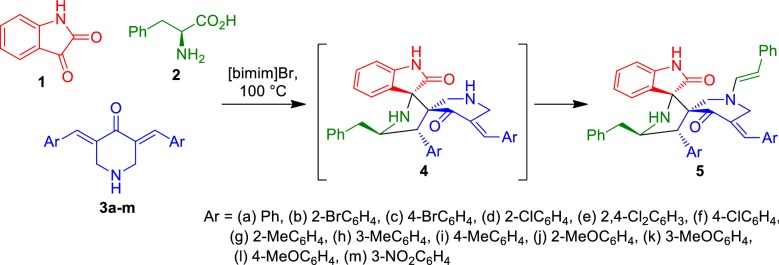

**Electronic supplementary material:**

The online version of this article (10.1186/s13065-018-0462-x) contains supplementary material, which is available to authorized users.

## Background

Cancer can be viewed as a group of related diseases that arise from abnormal cell growth and the loss of regulation of processes associated to programmed cell death via apoptosis [1]. Although cancer chemotherapy has progressed in major strides in recent years, there is still an unmet need for new anti-cancer agents with good potency, diminished toxicity and able to treat tumors that are resistant to currently known drugs [2].

Medicinal chemistry faces major challenges in designing new synthetic compounds with therapeutic importance. In particular, the therapy of complex and multifactorial diseases such as cancer may benefit from molecular design based on the multitarget ligand paradigm, i.e., by incorporation of various biologically active heterocyclic pharmacophores into a single molecule. The hybrid compounds thus generated, carrying more than one pharmacophoric entity and wherein each individual active unit may exert diverse modes of action, offer a new hope in the treatment of cancer.

**Fig. 1:**
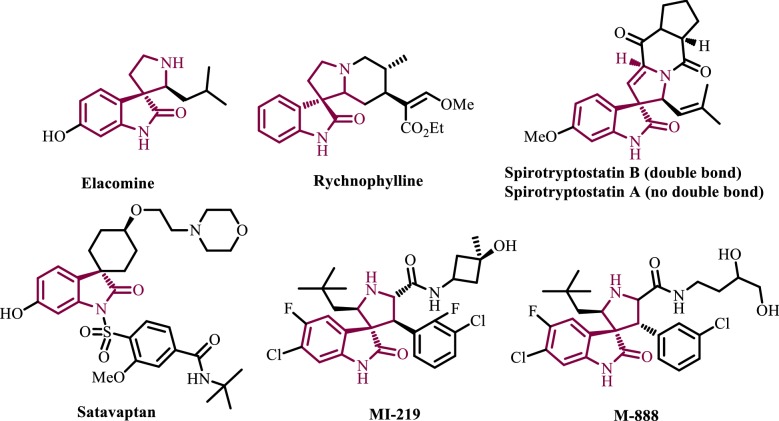
Representative biologically relevant natural spiro(oxindole-pyrrolidine) derivatives

One of the current trends in the discovery of lead compounds for drug discovery programs, that has been described as “escape from flatland”, is an increased three-dimensionality, involving the move from planar aromatic or heteroaromatic systems to others with a higher level of saturation. Such compounds are expected to interact more efficiently with binding pockets in proteins, which are three-dimensional in nature, and have better solubility, a crucial property in the process of drug development [3]. Spiro compounds are very attractive in this connection, since they are intrinsically three-dimensional and many bioactive natural products contain spirocyclic cores that can be assumed to have arisen in the course of evolution to allow better interaction with proteins [4]. In particular, spiro-oxindolepyrrolidine cores can be found in a variety of alkaloids [5], including elacomine, rhynchophylline and the spirotryprostatins, among many others. These compounds and many additional synthetic spirooxindolo-pyrrolidine derivatives (Fig. 1) have shown anticancer [6–8] and other important pharmacological activities [9–13]. 3-Arylmethylene-4-piperidone is another important heterocyclic scaffold present in several families of tumor-specific cytotoxins that display excellent apoptotic-inducing properties against a number of human cancer cell lines, being especially effective against colon cancers and leukemic cells [14].

In recent years, our research group has been involved in the synthesis [15–18] and biological evaluation [19–22] of spiroheterocyclic hybrids containing piperidin-4-one units, which were obtained through domino reaction sequences comprising a multicomponent 1,3-dipolar cycloaddition step. In continuation of our research interest in this area, we reasoned that the combination of the spirooxindole framework with pyrrolidine and piperidone motifs in a single molecule would be of interest in the context of anticancer drug discovery.

## Results and discussion

### Chemistry

**Scheme 1:**
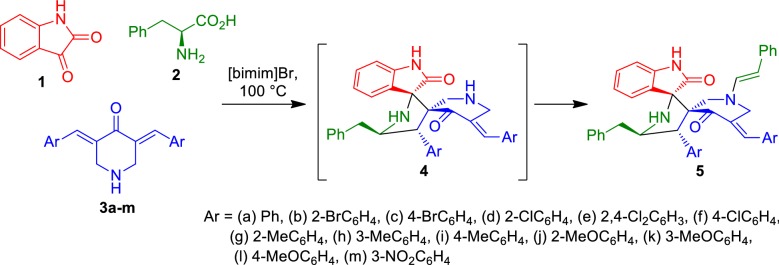
Synthesis of *N*-arylidenepiperidone tethered dispiropyrrolidine** 5**

**Scheme 2:**
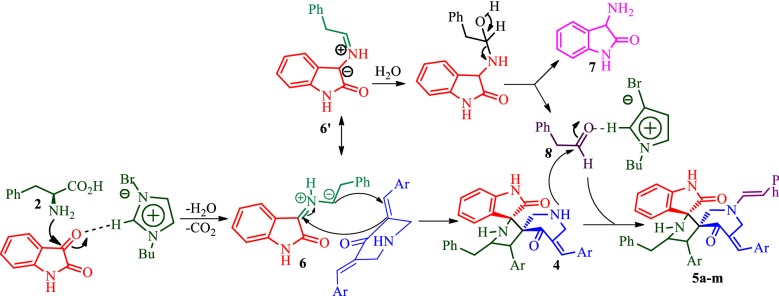
Plausible mechanism for the regio- and stereo selective product formation through a domino sequence

Synthetic methodology employed in the present work was based on the multicomponent 1,3-dipolar cycloaddition reaction strategy as summarized in Scheme 1, and involved a tandem process comprising the 1,3-dipolar cycloaddition reaction between bis-benzylidenepiperidinone and azomethine ylide **6**, generated in situ from isatin **1** and l-phenylalanine **2**, to afford spiroheterocycle **5**. This intermediate subsequently reacts with 2-phenylacetaldehyde, generated in situ in the course of the reaction mechanism (see Scheme 2 below) to afford the final *N*-substituted arylmethylidene piperidone tethered dispiropyrrolidines **5** through formation of an enamine reaction. Since l-phenylalanine, one of the reaction components, takes part at two different stages of the mechanism and with two different roles, this reaction can be regarded as an example of a rare chemo-differentiating ABCC′ multicomponent reaction [23].

Regarding solvent optimization, the reaction was initially performed with an equimolar mixture of (3*E*,5*E*)-3,5-bis(4-methylbenzylidene)piperidin-4-one, isatin and l-phenylalanine in methanol, which afforded the product **5a** in only 25% yield even after 10 h under reflux. The starting material **3** was still present in the reaction mixture, as evidenced by TLC. After verifying the participation of two molecules of phenylalanine, the same reaction was performed in 1:2:2.05 molar ratio and was found to be complete in 2 h (TLC), affording the product in good yield. The reaction was also attempted under reflux in different solvents or solvent mixtures, viz, dioxane, acetonitrile, dioxane/methanol (1:1 v/v) and toluene. In all these cases, compound **5a** was formed only in moderate yields even after long reaction times. As part of our interest in the use of ionic liquids to promote 1,3-dipolar cycloadditions [20, 22], we also examined the use of [bmim]Br as the reaction medium for the present reaction. An excellent yield of the product was obtained in a short reaction time, as shown in Table 1, all successive reactions were accomplished under these optimized reaction conditions (Table 2).

**Table 1:** Optimization of solvent for synthesis of spiroheterocyclic hybrids **5a**

Entry	Solvents	Time (h)	Yield (%)
1	Methanol	2	90
2	Dioxane	2	62
3	Acetonitrile	2	65
4	Dioxane: methanol	2	75
5	Toluene	2	55
6	[bmim]Br	1	95

**Table 2:**
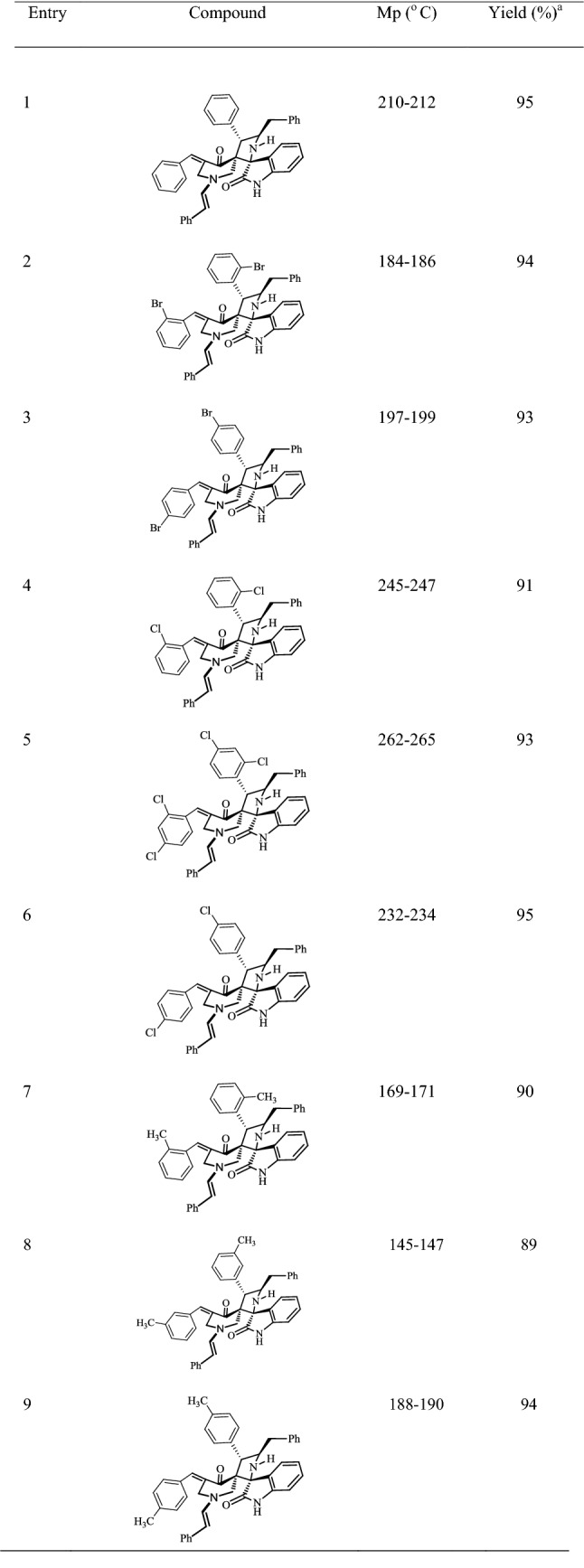

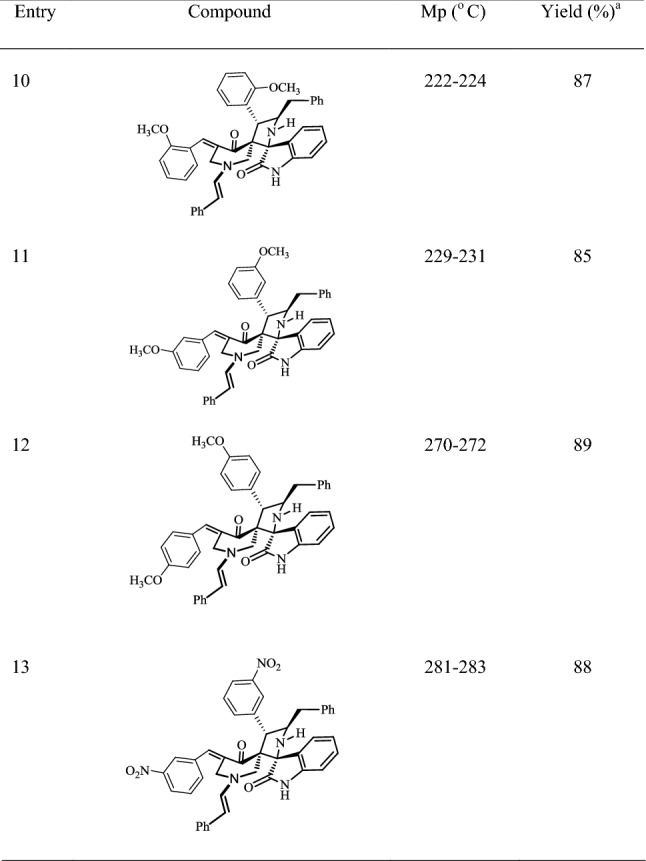
Structures, yields and melting points of compounds **5a**–**m**

^a^Isolated yield after column chromatography

**Fig. 2:**
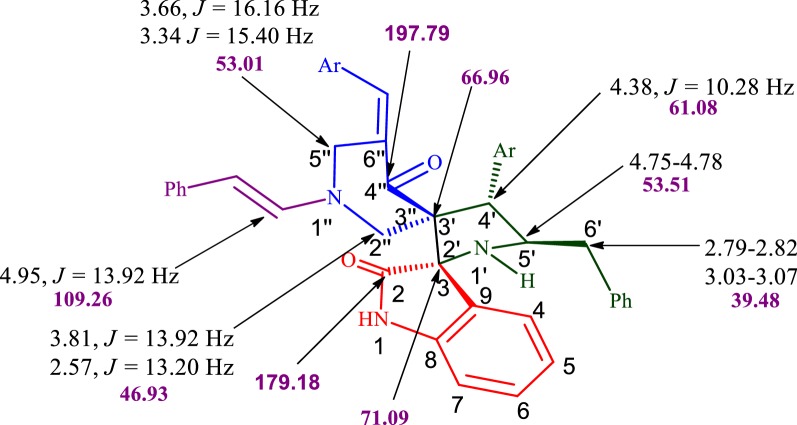
Assignments of selected signals of compound **5a**

The spiropyrrolidine derivatives **5a**–**m** thus obtained were characterized by one- and two-dimensional NMR experiments, as shown in Fig. 2 below for the representative case of **5a**. Its ^1^H NMR spectrum shows a doublet at 4.38 ppm (*J *= 10.3 Hz) for 4′-CH, i.e., the benzylic proton belonging to the pyrrolidine ring. This coupling constant value establishes the regiochemistry of the cycloaddition, since 4′-CH should give a singlet for the other possible regiomers arising from the cycloaddition. The multiplet found at 4.75–4.78 ppm, which was shown to be coupled to 4′-CH in the H,H-COSY experiment, was assigned to 5′-CH. The multiplets at 2.79–2.82 and 3.03–3.07 ppm were assigned to 6′-CH_2_ because they are coupled with 5′-CH. Again from COSY data, the doublets at 3.81 ppm (*J *= 13.5 Hz) and 2.57 ppm (*J *= 13.5 Hz) can be assigned to the 2″-CH_2_ protons, which were also correlated with the carbonyl group of piperidone moiety at 197.79 ppm, as shown by the HMQC experiment. The 7″-arylmethylene proton was observed as a doublet at δ 4.95 ppm (*J *=13.9 Hz). The signals at 71.09 and 66.96 ppm in the ^13^C-NMR spectrum of **5a** were attributed to C-3′ and C-2′, respectively, while those at 39.48, 46.93 and 53.01 ppm were assigned to the three methylene carbons (C-6′, C-2″ and C-6″) using DEPT-135 data (Additional file 1). Finally in the mass spectrum of **5a**, the presence of molecular ion peak at *m/z* = 627 (M^+^) and a comparison with a similar analogue reported by us earlier [24] confirms the proposed structure.

A feasible mechanism for the formation of compounds **5** is illustrated in Scheme 2. Initially, the azomethine ylide **6** generated in situ by the reaction of indoline-2,3-dione and l-phenylalanine via decarboxylative condensation. The intermediate **6** then adds regioselectively to one of the C=C bonds of arylidinepiperidone **3** furnish cycloadduct **4**. Simultaneously, the azomethine ylide **6**′ would be attacked by a molecule of water to furnish 2-phenylacetaldehyde **8** and 3-aminoindolin-2-one **7** as a by-product. Finally, the condensation of this aldehyde with the free secondary amino group in **4** would form the enamine group in compounds **5**.

### Cytotoxicity analysis

**Fig. 3:**
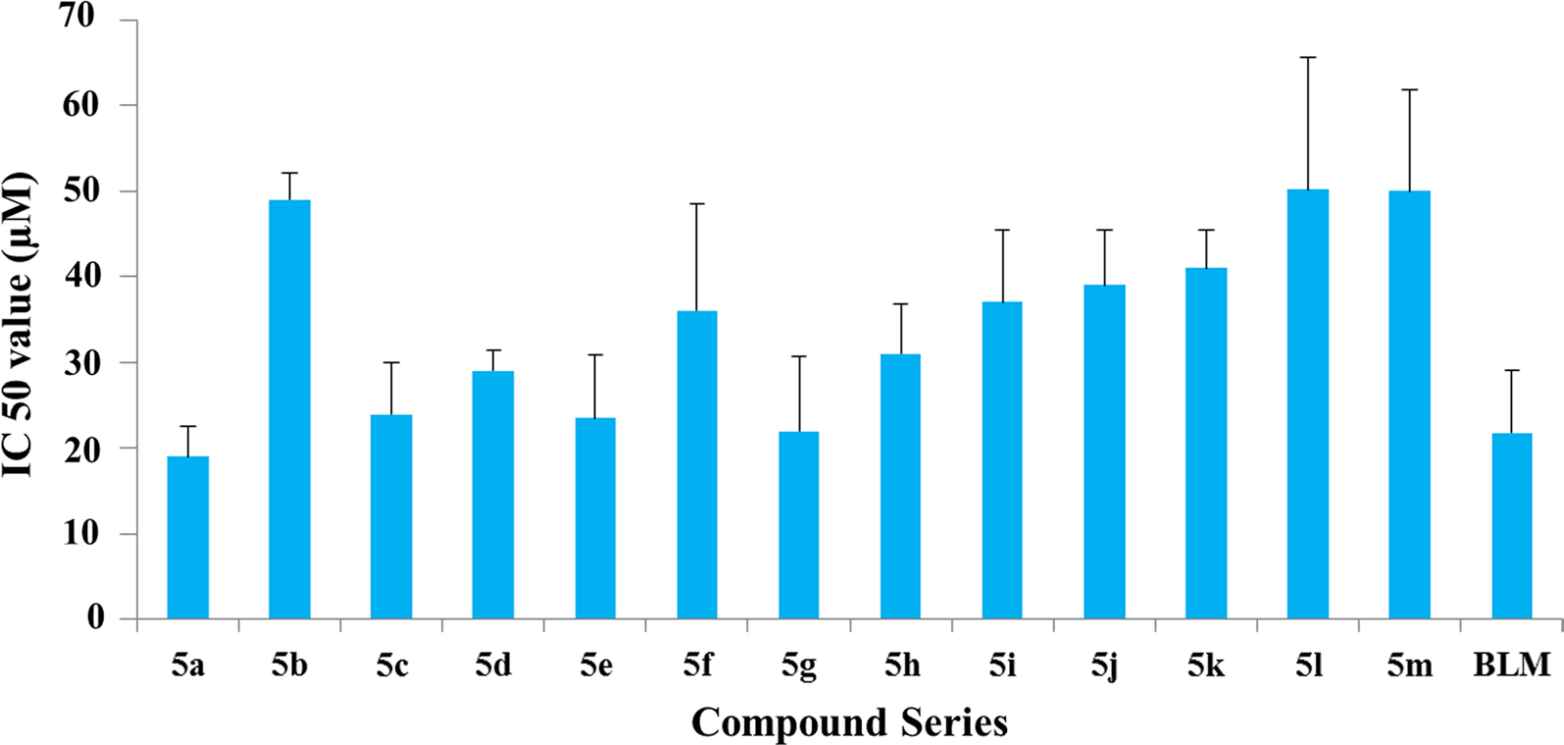
In vitro cytotoxicity analysis of synthesized compounds **5**(**a**–**m**) and bleomycin against FaDu hypopharyngeal cancer cells after 48 h incubation. The data are presented as the mean ± SD of four replicates each. The graph shows the IC_50_ values of the synthesized compounds and bleomycin

The cytotoxicity of compounds **5a**–**m** was assessed on FaDu hypopharyngeal tumor cells after their exposure to the compounds for 48 h, in comparison with the commercial anti-cancer drug bleomycin under identical conditions (Fig. 3). While **5l** and **5m** were inactive, the other compounds showed IC_50_ values in the 19–41 μM range (Additional file 1: Table S1), which are comparable to the one found for the standard anticancer drug bleomycin (IC_50_ = 21.8 ± 7.3). While the similar activities found for most compounds make it difficult to extract meaningful structure–activity relationships, the data obtained suggest that, with the exception of the Br derivatives, the most favourable position for substitution in the variable aryl ring is *ortho*- (e.g., **5g** vs **5i**, **5d** vs **5f**, **5h** vs **5i**). There does not seem to be any connection between activity and the electron-releasing or electron-withdrawing nature of the substituents, and in fact the three best compounds, which were comparable in terms of activity to the bleomycin positive control, are the parent unsubstituted system **5a**, the 2-methyl derivative **5g** and the 2,4-dichloro derivative **5e**.

### Quantitation of apoptotic cell percentage

**Fig. 4:**
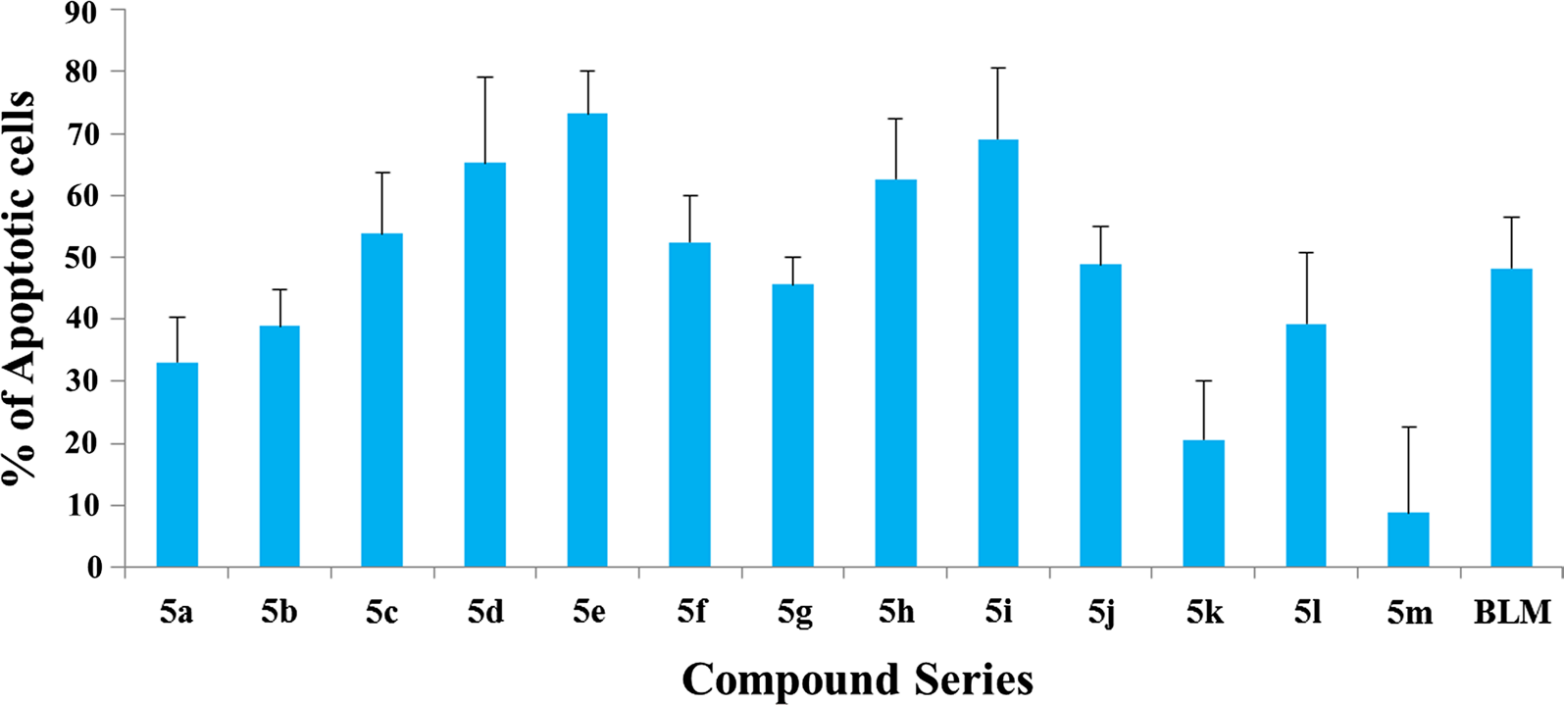
AO/EB dual staining data showing the response of FaDu hypopharyngeal cancer cells exposed to synthesized compounds **5**(**a**–**m**) and bleomycin at 48 h, in terms of apoptosis. The percentage of apoptotic cells is indicated by the histograms. The data shown are the means from triplicates

**Fig. 5:**
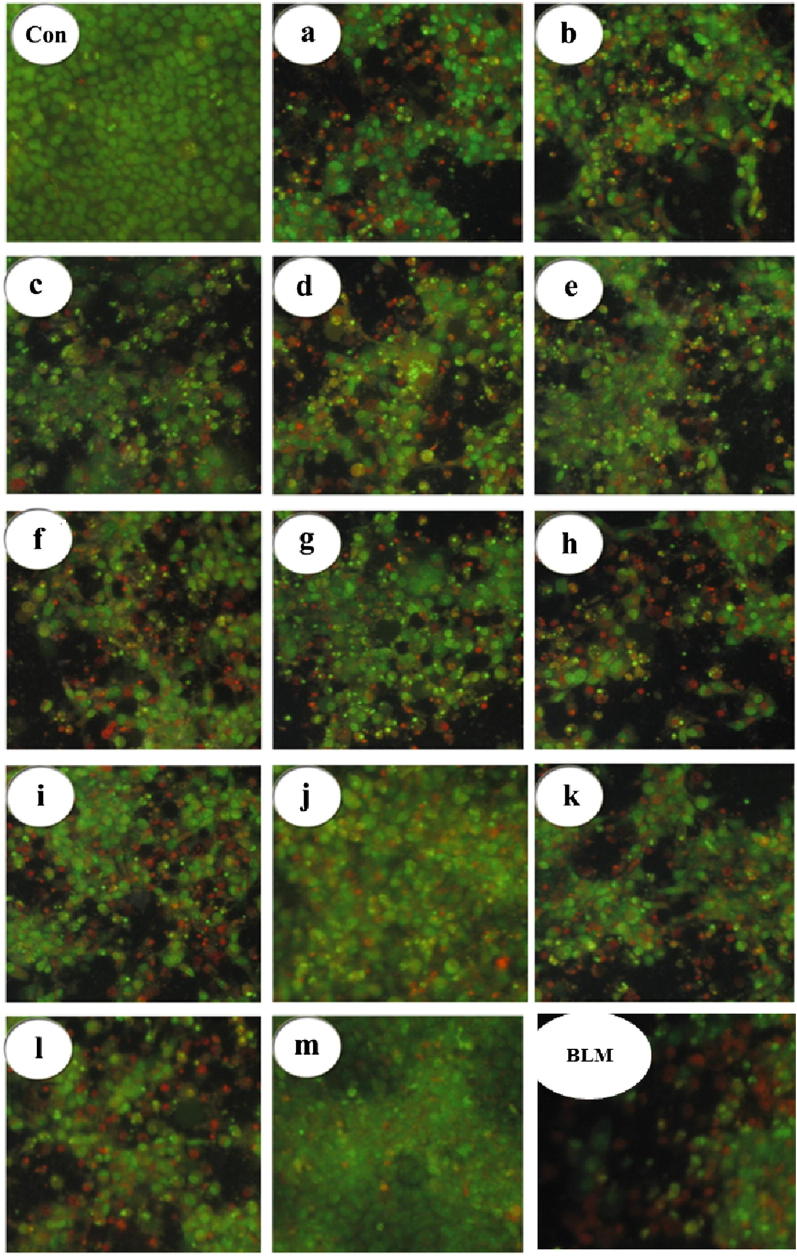
Cytological features of synthesized compounds **5**(**a**–**m**) and bleomycin-treated FaDu hypopharyngeal cancer cells (48 h). Magnification: ×400

We next studied the changes that our compounds induced in cell morphology. In order to study both cytoplasmic and nuclear morphological changes, we carried out a dual staining with acridine orange and ethidium bromide. The cancer cells were treated with our compounds at their IC_50_ concentrations for 48 h. The observed morphological changes could be classified as follows: (i) viable cells had shining nuclei, evenly green in color, and displayed a highly organized structure; (ii) early apoptotic cells showed shining nuclei, yellow-green in color, with nuclear chromatin that was crescent-shaped and condensed or fragmented (iii) late apoptotic cells displayed shining nuclei, orange to red in color, with chromatin condensation and fragmentation; and (iv) necrotic cells had orange to red shining nuclei and their volume was increased (Fig. 4). The early response observed following treatment with our compounds **5** was death by apoptosis, and the surviving cells succumbed to necrosis on prolonged treatment. Our findings indicate that the ability of compounds **5a**–**m** to induce apoptosis in FaDu cells had a good correlation to their cytotoxicity values, as shown in Fig. 5.

## Conclusion

A one-pot protocol has been developed in [bmim]Br for the construction of spiropyrrolidine tethered piperidone heterocyclic hybrids **5** from simple starting materials that involves the generation of novel structurally interesting *N*-arylidenepiperidone tethered spirooxindolopyrrolidine **5a**–**m** in good to excellent yields by creation of four new bonds and four adjacent stereocenters in a single operation. This four-component process involves the generation of an azomethine ylide, a regio- and diastereoselective 1,3-dipolar cycloaddition an enamine-formation reaction. These compounds were evaluated for their cytotoxicity against FaDu hypopharyngeal tumor cells and it was observed that most of them exhibited a cytotoxicity similar to the one found for the standard anticancer drug bleomycin, with two compounds (**5a** and **5g**) being slightly more potent than the reference drug. In addition, the compounds were shown to induce apoptosis in the same cancer cell model.

## Experimental

### General methods

The melting points were measured using open capillary tubes and are uncorrected. ^1^H, ^13^C and 2D NMR spectra were recorded on a JEOL 400 MHz instrument. Elemental analyses were carried out on a Perkin Elmer 2400 Series II Elemental CHNS analyser. Mass spectra were performed on JEOL-DX303 HF mass spectrometer.

### General procedure for synthesis of dispiropyrrolidines fused piperidinone heterocyclic hybrids, **5**(**a**–**m**)

The suitable arylimethylenepiperidin-4-one (1.36 mmol), isatin (2.72 mmol) and l-Phenylalanine (2.72 mmol) in [bmim]Br (3 mL) was heated while stirred for 1 h at 100 °C. After the reaction completion, EtOAc (2 × 5 mL) was added and the resulting mixture was stirred for an additional time of 10 min. The EtOAc layer was separated and the solvent was evaporated. The residue was recrystallized from ethanol to furnish compounds **5**.

#### Dispiropyrrolidine tethered piperidinone heterocyclic hybrid (**5a**)

^1^H NMR: *δ*/ppm 4.95 (1H, d, *J *= 4.92 Hz), 4.75–4.78 (1H, m), 4.38 (1H, d, *J *= 10.28 Hz), 3.81 (1H, d, *J *= 13.92 Hz), 3.66 (1H, d, *J *= 16.16 Hz), 3.34 (1H, d, *J *= 15.40 Hz), 3.03–3.07 (1H, m), 2.79–2.82 (1H, m), 2.57 (1H, d, *J *= 13.20 Hz), 6.61–6.64 (2H, m), 6.89–7.48 (24H, m, Ar), 7.68 (1H, s, NH); ^13^C NMR: *δ*_*C*_/ppm 39.48, 46.93, 53.01, 53.51, 61.24, 66.96, 71.09, 100.03, 109.26, 122.30, 124.09, 124.18, 126.37, 126.69, 127.23, 127.85, 128.46, 128.62, 128.64, 128.72, 129.27, 129.35, 129.42, 130.30, 130.69, 134.62, 137.26, 138.53, 138.64, 138.78, 139.15, 141.01, 179.18, 197.79. MS: *m/z* 627 (M^+^). Anal.Calcd for C_43_H_37_N_3_O_2_: C, 82.27; H, 5.94; N, 6.69. Found: C, 82.39; H, 5.81; N, 6.57.

#### Dispiropyrrolidine tethered piperidinone heterocyclic hybrid (**5b**)

^1^H NMR: *δ*/ppm 4.88–4.91 (1H, m), 4.57–4.62 (2H, m), 3.58 (1H, d, *J* = 16.16 Hz), 3.21 (1H, d, *J *= 13.92 Hz), 3.00–3.08 (1H, m), 2.97–2.99 (1H, m), 2.74 (1H, d, *J *= 15.4 Hz), 6.35 (1H, d, *J *= 13.92 Hz), 6.65 (1H, d, *J *= 8.04 Hz), 6.92–7.69 (22H, m, Ar), 7.95 (1H, s, NH); ^13^C NMR: *δ*/ppm 39.58, 46.92, 53.24, 53.57, 61.29, 65.45, 73.57, 100.13, 109.16, 122.39, 123.75, 125.18, 126.14, 126.26, 126.74, 127.28, 127.89, 128.30, 128.40, 129.26, 129.34, 129.41, 129.44, 130.38, 130.78, 130.86, 132.82, 132.83, 135.07, 135.35, 136.28, 137.35, 138.59, 138.69, 138.81, 139.21 141.17, 179.24, 197.81. MS: m/z 785 (M^+^). Anal.Calcd for H_35_Br_2_N_3_O_2_: C, 65.74; H, 4.49; N, 5.35; Found: C, 65.86; H, 4.61; N, 5.47.

#### Dispiropyrrolidine tethered piperidinone heterocyclic hybrid (**5c**)

^1^H NMR: *δ*/ppm 4.94 (1H, d, *J *= 13.92 Hz), 4.66–4.70 (1H, m), 4.30 (1H, d, *J *= 10.24 Hz), 3.74 (1H, d, *J *= 13.96 Hz), 3.65 (1H, d, *J *= 15.4 Hz), 3.27 (1H, d, *J *= 15.76 Hz), 2.98–3.03 (1H, dd, *J *= 13.92, 2.96 Hz), 2.77–2.82 (1H, m, 13.92, 8.08 Hz), 2.56 (1H, d, *J *= 13.2 Hz), 6.59–6.64 (2H, m), 6.89–7.49 (22H, m, Ar); ^13^C NMR: *δ*/ppm 39.52, 46.96, 52.94, 53.06, 61.53, 66.73, 71.14, 100.57, 109.37, 121.28, 122.31, 124.17, 124.40, 126.48, 126.55, 126.56, 128.24, 128.50, 128.64, 129.28, 129.41, 131.13, 131.51, 131.62, 131.84, 131.95, 133.35, 136.30, 137.91, 138.21, 138.29, 138.48, 141.01, 179.22, 197.94. MS: m/z 785 (M^+^). Anal.Calcd for C_43_H_35_Br_2_N_3_O_2_: C, 65.74; H, 4.49; N, 5.35;. Found: C, 65.86; H, 4.62; N, 5.47.

#### Dispiropyrrolidine tethered piperidinone heterocyclic hybrid (**5d**)

^1^H NMR: *δ*/ppm 4.88–4.92 (1H, m), 4.62–4.65 (2H, m), 3.59 (1H, d, *J *= 16.16 Hz), 3.26 (1H, d, *J *= 13.92 Hz), 2.96–3.10 (2H, m), 2.77–2.86 (2H, m), 6.38 (1H, d, *J *= 13.92 Hz), 6.64 (1H, d, *J *= 7.32 Hz), 6.89–7.42 (22H, m, Ar), 7.76 (1H, s); ^13^C NMR: *δ*/ppm 40.53, 46.15, 51.89, 52.62, 62.90, 65.46, 73.28, 98.25, 109.78, 122.55, 123.84, 126.18, 126.25, 126.31, 126.52, 127.52, 127.09, 127.69, 128.09, 128.36, 128.61, 129.18, 129.29, 130.03, 130.36, 130.50, 130.57, 132.94, 133.11, 135.07, 135.80, 135.92, 136.20, 137.94, 138.51, 139.08, 141.16, 177.48, 200.01. MS: m/z 696 (M^+^). Anal. Calcd for C_43_H_35_Cl_2_N_3_O_2_: C, 74.13; H, 5.06; N, 6.03; Found: C, 74.24; H, 5.17; N, 6.15.

#### Dispiropyrrolidine tethered piperidinone heterocyclic hybrid (**5e**)

^1^H NMR: *δ*/ppm 4.82–4.87 (1H, m), 4.58–4.67 (2H, m), 3.55–60 (1H, d, *J *= 16.16 Hz), 3.23 (1H, d, *J *= 13.92 Hz), 2.96–3.01 (2H, m), 2.77–2.81 (2H, m), 6.33 (1H, d, *J *= 13.92 Hz), 6.66 (1H, d, *J *= 7.36 Hz) 6.90–7.50 (20H, m, Ar), 7.58 (1H, s, NH); ^13^C NMR: *δ*/ppm 39.54, 46.92, 53.17, 53.65, 61.36, 67.01, 71.18, 100.12, 109.30, 122.31, 123.90, 124.18, 124.20, 126.04, 126.42, 126.70, 127.05, 127.91, 128.27, 128.39, 128.63, 129.09, 129.58, 129.90, 129.96, 130.66, 131.52, 133.27, 133.46, 134.91, 135.75, 135.81, 137.57, 138.11, 138.69, 139.24, 141.11, 179.26, 197.64. MS: m/z 765 (M^+^). Anal.Calcd for C_43_H_33_Cl_4_N_3_O_2_: C, 67.46; H, 4.34; N, 5.49. Found: C, 67.58; H, 4.47; N, 5.62.

#### Dispiropyrrolidine tethered piperidinone heterocyclic hybrid (**5f**)

^1^H NMR: *δ*/ppm 4.94 (1H, d, *J *= 13.96 Hz), 4.67–4.68 (1H, m), 4.32 (1H, d, *J *= 10.4 Hz), 3.75 (1H, d, *J *= 13.16 Hz), 3.62 (1H, d, *J *= 16.12 Hz), 3.29 (1H, d, *J *= 16.16 Hz), 2.97–3.00 (m, 1H), 2.77–2.80 (1H, m), 2.55 (1H, d, *J *= 13.92 Hz), 6.56–6.64 (2H, m), 6.88–7.39 (22H, m, Ar), 7.80 (1H, s, NH); ^13^C NMR: *δ*/ppm 39.55, 46.98, 52.88, 52.94, 61.47, 66.73, 71.12, 100.55, 109.40, 122.29, 124.14, 124.37, 126.47, 126.63, 127.68, 128.47, 128.62, 128.87, 128.99, 129.33, 129.38, 129.41, 131.05, 131.46, 132.95, 133.10, 135.57, 135.80, 137.83, 138.21, 138.32, 138.51, 141.07, 179.24, 197.50. MS: m/z 696 (M^+^). Anal.Calcd for C_43_H_35_Cl_2_N_3_O_2_: C, 74.13; H, 5.06; N, 6.03 Found: C, 74.24; H, 5.18; N, 6.15.

#### Dispiropyrrolidine tethered piperidinone heterocyclic hybrid (**5g**)

^1^H NMR: *δ*/ppm 4.91–4.93 (1H, m), 4.70 (1H, d, *J *= 13.92 Hz), 4.49 (1H, d, *J *= 10.28 Hz), 3.53–3.59 (2H, m), 2.97–3.09 (1H, m), 2.83–2.89 (2H, m), 2.63 (1H, d, *J *= 13.96 Hz), 2.23 (3H, s), 2.21 (3H, s), 6.48 (1H, d, *J *= 14.64 Hz), 6.65 (1H, d, *J *= 7.32 Hz), 6.76 (1H, d, *J *= 8.08 Hz), 6.92–7.76 (21H, m, Ar), 7.65 (1H, s, NH); ^13^C NMR: *δ*/ppm 20.06, 20.99, 40.12, 46.22, 50.46, 53.21, 63.16, 65.67, 72.53, 98.54, 109.58, 122.38, 123.87, 125.75, 125.81, 126.30, 126.38, 126.40, 126.51, 126.87, 128.43, 128.60, 128.65, 128.81, 129.15, 129.29, 130.41, 131.54, 133.67, 136.04, 137.86, 138.01, 138.24, 138.36, 138.43, 138.95, 139.08, 141.22, 177.91, 199.89. MS: m/z 655 (M^+^). Anal.Calcd for C_45_H_41_N_3_O_2_; C, 82.41; H, 6.30; N, 6.41; Found: C, 82.54; H, 6.42; N, 6.53.

#### Dispiropyrrolidine tethered piperidinone heterocyclic hybrid (**5h**)

^1^H NMR: *δ*/ppm 4.95 (1H, d, *J *= 13.96 Hz), 4.72–4.76 (m, 1H), 4.34 (1H, d, *J *= 11.0 Hz), 3.82 (1H, d, *J *= 13.92 Hz), 3.66 (1H, d, *J *= 17.6 Hz), 3.36 (d, *J *= 16.12 Hz, 1H), 3.04–3.07 (1H, m), 2.75–2.81 (1H, m), 2.58 (1H, d, *J *= 13.2 Hz), 2.36 (3H, s), 2.34 (3H, s), 6.60–6.64 (2H, m), 6.74–7.34 (22H, m, Ar), 7.72 (1H, s, NH); ^13^C NMR: *δ*/ppm 20.03, 20.98, 40.16, 46.29, 50.42, 53.19, 63.20, 65.73, 72.56, 98.58, 109.63, 122.41, 123.91, 125.79, 126.32, 126.45, 126.54, 126.89, 126.92, 128.41, 128.64, 128.86, 129.18, 129.32, 129.41, 129.44, 129.58, 130.47, 131.55, 133.68, 136.09, 137.88, 138.03, 138.25, 138.41, 138.99, 139.08, 141.23, 177.96, 199.91. MS: m/z 655 (M^+^). Anal.Calcd for C_45_H_41_N_3_O_2_; C, 82.41; H, 6.30; N, 6.41; Found: C, 82.54; H, 6.42; N, 6.51.

#### Dispiropyrrolidine tethered piperidinone heterocyclic hybrid (**5i**)

^1^H NMR: *δ*/ppm 4.96 (1H, d, *J *= 13.96 Hz), 4.71–4.75 (1H, m), 4.35 (1H, d, *J *= 10.28 Hz), 3.79 (1H, d, *J* = 13.96 Hz), 3.67 (1H, d, *J *= 15.4 Hz), 3.35 (d, *J *= 16.12 Hz), 3.03–3.06 (1H, m), 2.76–2.78 (1H, m), 2.60 (1H, d, *J *= 13.92 Hz), 2.36 (3H, s), 2.34 (3H, s), 6.60 (2H, m), 6.64 (1H, s), 6.84–7.35 (21H, m, Ar), 7.64 (1H, s, NH); ^13^C NMR: *δ*/ppm 21.20, 21.55, 39.40, 47.09, 53.03, 53.16, 61.22, 66.83, 71.12, 99.94, 109.19, 122.24, 124.06, 124.11, 126.33, 126.69, 127.96, 128.44, 128.60, 129.04, 129.19, 129.37, 129.41, 129.91, 130.12, 130.50, 131.87, 134.17, 136.81, 138.62, 138.76, 138.85, 139.14, 139.85, 141.02, 179.33, 197.84. MS: m/z 655 (M^+^). Anal.Calcd for C_45_H_41_N_3_O_2_; C, 82.41; H, 6.30; N, 6.41; Found: C, 82.53; H, 6.44; N, 6.52.

#### Dispiropyrrolidine tethered piperidinone heterocyclic hybrid (**5j**)

^1^H NMR: *δ*/ppm 5.00–5.02 (1H, m), 4.66 (1H, d, *J *= 13.96 Hz), 4.47 (1H, d, *J *= 10.28 Hz), 3.86 (3H, s), 3.82–3.74 (4H, m), 3.67 (1H, d, *J *= 16.12 Hz), 3.26 (1H, d, *J *= 13.96 Hz), 3.06–3.08 (1H, m), 2.87–2.93 (1H, m), 2.80 (1H, d, *J *= 13.92 Hz), 6.45 (1H, m, *J *= 13.92 Hz), 6.61 (1H, d, *J *= 8.08 Hz), 6.76–7.34 (22H, m, Ar), 7.73 (1H, s, NH); ^13^C NMR: *δ*/ppm 40.48, 46.40, 48.37, 52.25, 55.01, 55.45, 60.49, 65.14, 72.71, 97.48, 109.58, 110.71, 120.20, 120.81, 122.24, 123.55, 123.68, 126.38, 126.43, 126.56, 126.88, 126.95, 127.79, 128.37, 128.56, 128.62, 128.76, 128.98, 129.12, 130.24, 130.92, 131.58, 134,11, 138.27, 139.26, 139.38, 139.47, 141.27, 179.92, 200.45. MS: m/z 687 (M^+^). Anal.Calcd for C_45_H_41_N_3_O_4_; C, 78.58; H, 6.01; N, 6.11; Found: C, 78.70; H, 6.13; N, 6.21.40.48, 46.40, 48.37, 52.25, 55.01, 55.45, 60.49, 65.14, 72.71, 97.48,

#### Dispiropyrrolidine tethered piperidinone heterocyclic hybrid (**5k**)

^1^H NMR: *δ*/ppm 5.01–5.04 (1H, m), 4.68 (1H, d, *J *= 13.96 Hz), 4.49 (1H, d, *J *= 10.28 Hz), 3.84 (3H, s), 3.80–3.82 (1H, m), 3.79 (3H, s), 3.65 (1H, d, *J *= 16.12 Hz), 3.28 (1H, d, *J *= 13.96 Hz), 3.05–3.07 (1H, m), 2.86–2.92 (1H, m), 2.82 (1H, d, *J *= 13.92 Hz), 6.44 (1H, m, *J *= 13.92 Hz), 6.62 (1H, d, *J *= 8.08 Hz), 6.77–7.38 (22H, m, Ar),7.71 (1H, s, NH); ^13^C NMR: *δ*/ppm 40.45, 46.40, 48.39, 52.21, 55.09, 60.52, 65.17, 72.73, 97.50, 109.59, 110.74, 120.23, 120.78, 122.21, 123.50, 123.61, 126.39, 126.41, 126.44, 126.54, 126.58, 126.80, 126.92, 126.92, 127.80, 128.41, 128.61, 128.91, 129.14, 130.27, 130.93, 131.55, 134,12, 138.29, 139.30, 139.39, 139.46, 141.32, 179.89, 199.82. MS: m/z 687 (M^+^). Anal.Calcd for C_45_H_41_N_3_O_4_; C, 78.58; H, 6.01; N, 6.11; Found: C, 78.71; H, 6.12; N, 6.23.

#### Dispiropyrrolidine tethered piperidinone heterocyclic hybrid (**5l**)

^1^H NMR: *δ*/ppm 4.99–5.02 (1H, m), 4.66 (1H, d, *J *= 13.96 Hz), 4.46 (1H, d, *J *= 10.28 Hz), 3.83 (3H, s), 3.80–3.82 (1H, m), 3.79 (3H, s), 3.64 (1H, d, *J *= 16.12 Hz), 3.29 (1H, d, *J *= 13.96 Hz), 3.04–3.08 (1H, m), 2.87–2.93 (1H, m), 2.83 (1H, d, *J *= 13.92 Hz), 6.46 (1H, m, *J *= 13.92 Hz), 6.61 (1H, d, *J *= 8.08 Hz), 6.78–7.39 (22H, m, Ar), 7.73 (1H, s, NH); ^13^C NMR: *δ*/ppm 40.42, 46.41, 48.40, 52.29, 55.11, 60.46, 65.22, 72.78, 97.80, 109.60, 110.76, 120.27, 120.79, 122.44, 123.93, 124.25, 124.50, 126.43, 126.58, 128.22, 128.31, 128.61, 129.28, 129.43, 129.80, 132.84, 134.67, 135.59, 136.41, 137.62, 138.07, 139.62, 140.81, 141.24, 148.30, 179.85, 199.94. MS: m/z 687 (M^+^). Anal.Calcd for C_45_H_41_N_3_O_4_; C, 78.58; H, 6.01; N, 6.11; Found: C, 78.72; H, 6.11; N, 6.22.

#### Dispiropyrrolidine tethered piperidinone heterocyclic hybrid (**5m**)

^1^H NMR: *δ*/ppm ^1^H NMR: *δ*/ppm 4.95 (1H, d, *J *= 14.68 Hz), 4.74–4.78 (1H, m), 4.42 (1H, d, *J *= 10.28 Hz), 3.71–3.76 (1H, m), 3.62 (1H, d, *J *= 16.12 Hz), 3.44–3.48 (1H, m), 3.32 (1H, d, *J *= 16.16 Hz), 2.84–2.88 (m, 1H), 2.52 (1H, d, *J *= 13.2 Hz), 6.71 (1H, d, *J *= 8.08 Hz), 6.93–7.48 (22H, m, Ar), 7.87 (1H, s, NH); ^13^C NMR: *δ*/ppm 40.42, 46.91, 53.45, 53.54, 55.12, 61.28, 66.97, 71.18, 100.23, 109.28, 122.35, 124.11, 124.21, 126.40, 126.74, 127.29, 127.37, 127.51, 127.68, 127.89, 128.51, 128.68, 128. 69, 128.71, 128.77, 129.29, 129.33, 129.39, 130.32, 130.71, 134.66, 137.31, 138.67, 138.74, 138.78, 139.19, 141.11, 179.19, 197.81. MS: m/z 717 (M^+^). Anal.Calcd for C_43_H_35_N_5_O_6_: 71.95; H, 4.91; N, 9.76; Found: 71.87; H, 4.99; N, 9.88.

## Additional file

**Additional file 1.** Experiment details and NMR spectra. **Table S1.** IC_50_ values of spiropyrrolidines **5** against FaDu hypopharyngeal cancer cells. **Figure S1.** ^1^H NMR spectrum of **5a**. **Figure S2.** Expanded ^1^H NMR spectrum of **5a**. **Figure S3.** ^13^C NMR spectrum of **5a**. **Figure S4.** DEPT-135 spectrum of **5a**. **Figure S5.** ^1^H, ^1^H-COSY spectrum of **5a**.

## Abbreviations

[bmim]Br: 1-butyl 3-methylimidazolium bromide
TLC: thin layer Chromatography
EtOAc: ethyl acetate
NMR: nuclear magnetic resonance
COSY: correlated spectroscopy
DEPT: distortion less enhancement by polarization transfer
EI-MS: electron ionization mass spectrometry
m/z: mass–charge ratio
