# Supplementary material for: Regio and stereoselective synthesis of anticancer spirooxindolopyrrolidine embedded piperidone heterocyclic hybrids derived from one-pot cascade protocol
Source: Chem Cent J. 2018 Sep 1;12:95. doi: 10.1186/s13065-018-0462-x (PMC6119554; doi:10.1186/s13065-018-0462-x)
Supplement: Supplementary file 1 — Additional file 1. Experiment details and NMR spectra. Table S1. IC50 values of spiropyrrolidines 5 against FaDu hypopharyngeal cancer cells. Figure S1. 1H NMR spectrum of 5a. Figure S2. Expanded 1H NMR spectrum of 5a. Figure S3. 13C NMR spectrum of 5a. Figure S4. DEPT-135 spectrum of 5a. Figure S5. 1H, 1H-COSY spectrum of 5a. [file 13065_2018_462_MOESM1_ESM.docx]

**Additional file 1**

**Regio and stereoselective synthesis of anticancer**

**spirooxindolopyrrolidine embedded piperidone heterocyclic hybrids derived from one-pot cascade protocol**

Natarajan Arumugam^1*^, Abdulrahman I. Almansour^1^, Raju Suresh Kumar^1^, Dhaifallah M. Al-thamili^1^, Govindasami Periyasami,^1^ V. S Periasamy,^2^ Jegan Athinarayanan,^2^ Ali A. Alshatwi,^2^ S. M. Mahalingam,^3^ J. Carlos Menéndez^4^

**___________________________________________________________________________________________________________**

| **S. No.** | **Figure** | **Page No.** |
| --- | --- | --- |
| **1** | IC_50_ values of spiropyrrolidines **5** against FaDu hypopharyngeal cancer cells | **S2** |
| **2** | ^1^H NMR spectrum of **4a** | **S3** |
| **3** | Expanded ^1^H NMR spectrum of **4a** | **S4** |
| **4** | ^13^C NMR spectrum of **4a** | **S5** |
| **5** | DEPT-135 NMR spectrum of **4a** | **S6** |
| **6** | ^1^H,^1^H-COSY spectrum of **4a** | **S7** |

**Table S1**. IC_50_ values of spiropyrrolidines **5** against FaDu hypopharyngeal cancer cells

| Entry | Compound | Ar | IC_50_, μM  (48 h incubation) |
| --- | --- | --- | --- |
| 1 | **5a** | Ph | 19.0 |
| 2 | **5b** | 2-BrC_6_H_4_ | 49.0 |
| 3 | **5c** | 4-BrC_6_H_4_ | 24.0 |
| 4 | **5d** | 2-ClC_6_H_4_ | 29.0 |
| 5 | **5e** | 2,4-Cl_2_C_6_H_3_ | 23.5 |
| 6 | **5f** | 4-ClC_6_H_4_ | 36.0 |
| 7 | **5g** | 2-MeC_6_H_4_ | 22.0 |
| 8 | **5h** | 3-MeC_6_H_4_ | 31.0 |
| 9 | **5i** | 4-MeC_6_H_4_ | 37.0 |
| 10 | **5j** | 2-MeOC_6_H_4_ | 39.0 |
| 11 | **5k** | 3-MeOC_6_H_4_ | 41.0 |
| 12 | **5l** | 4-MeOC_6_H_4_ | >50 |
| 13 | **5m** | 3-NO_2_C_6_H_4_ | >50 |
| 14 | BLM | -- | 21.8 |

| 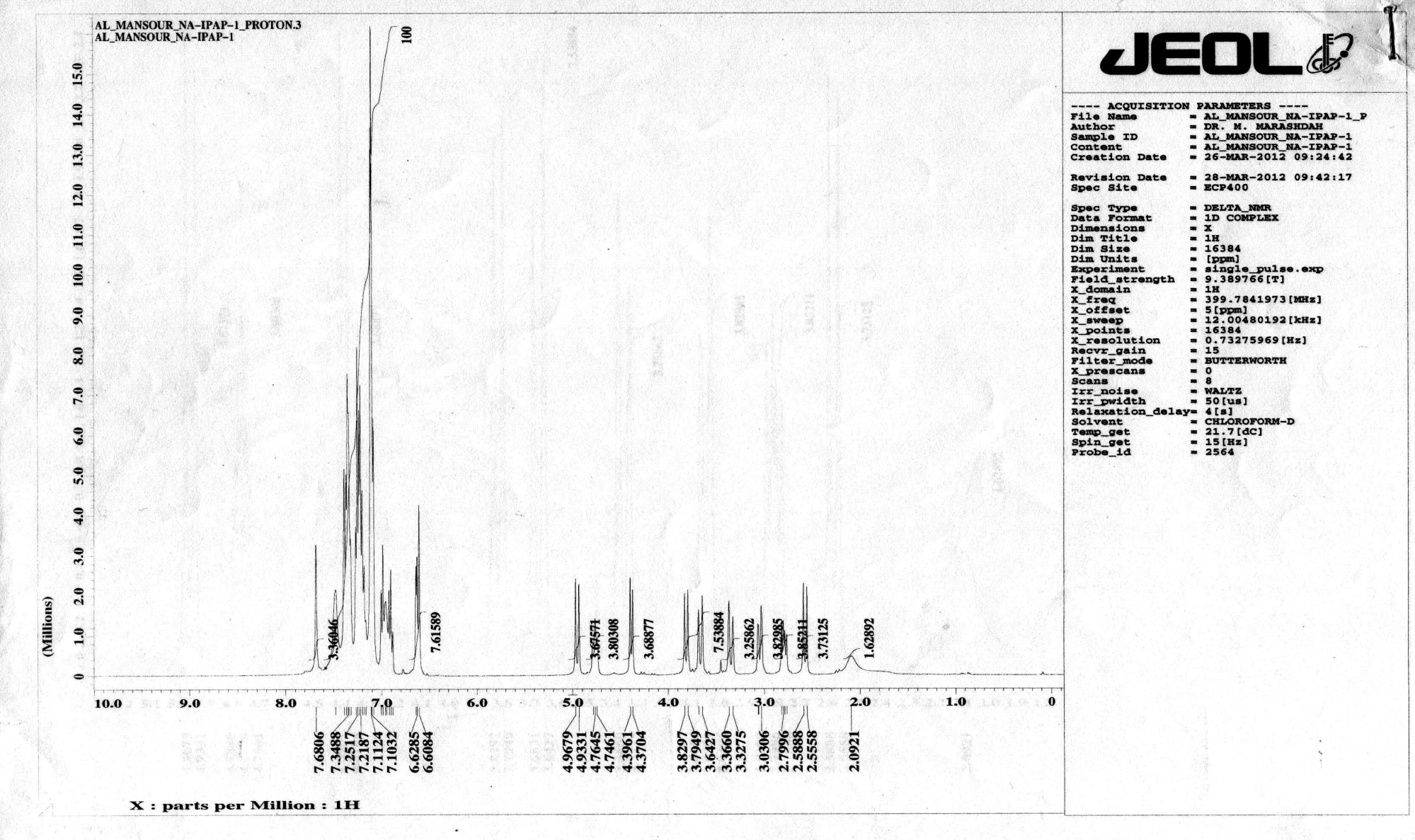 |
| --- |

**Figure S1**. ^1^H NMR spectrum of 5**a**

| 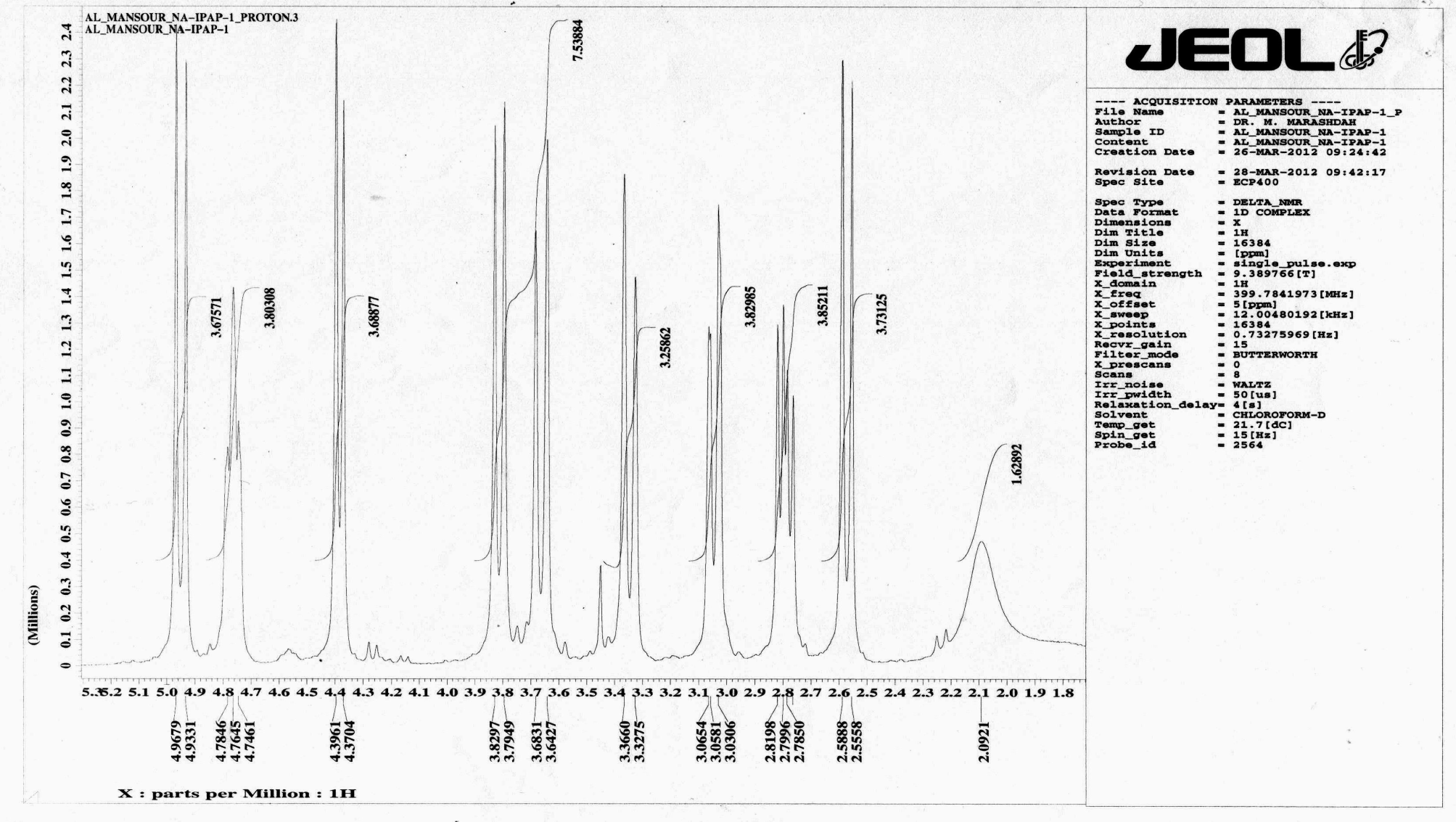 |
| --- |

**Figure S2**. Expanded ^1^H NMR spectrum of 5**a**

| 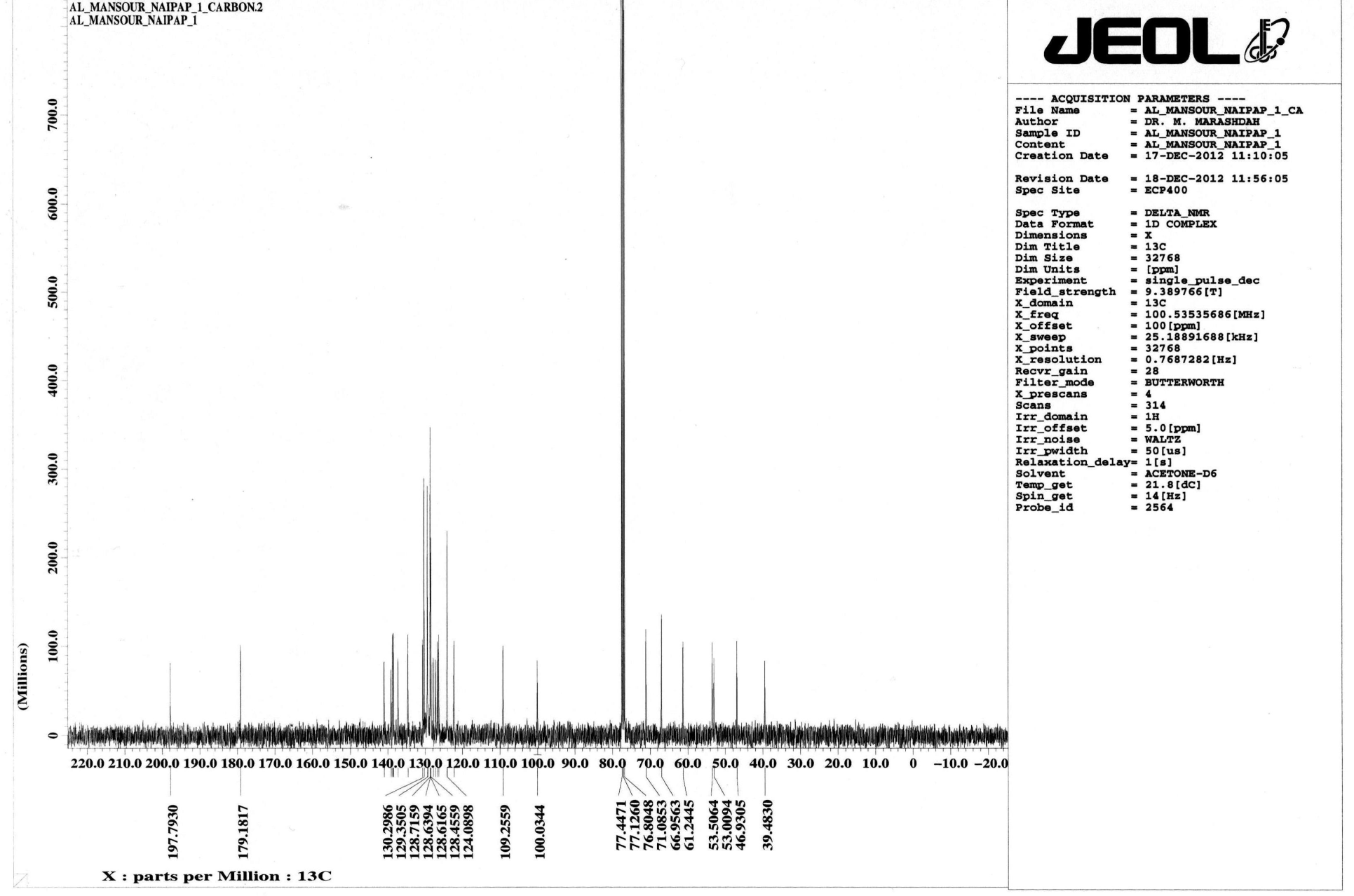 |
| --- |

**Figure S3**. ^13^C NMR spectrum of 5**a**

| 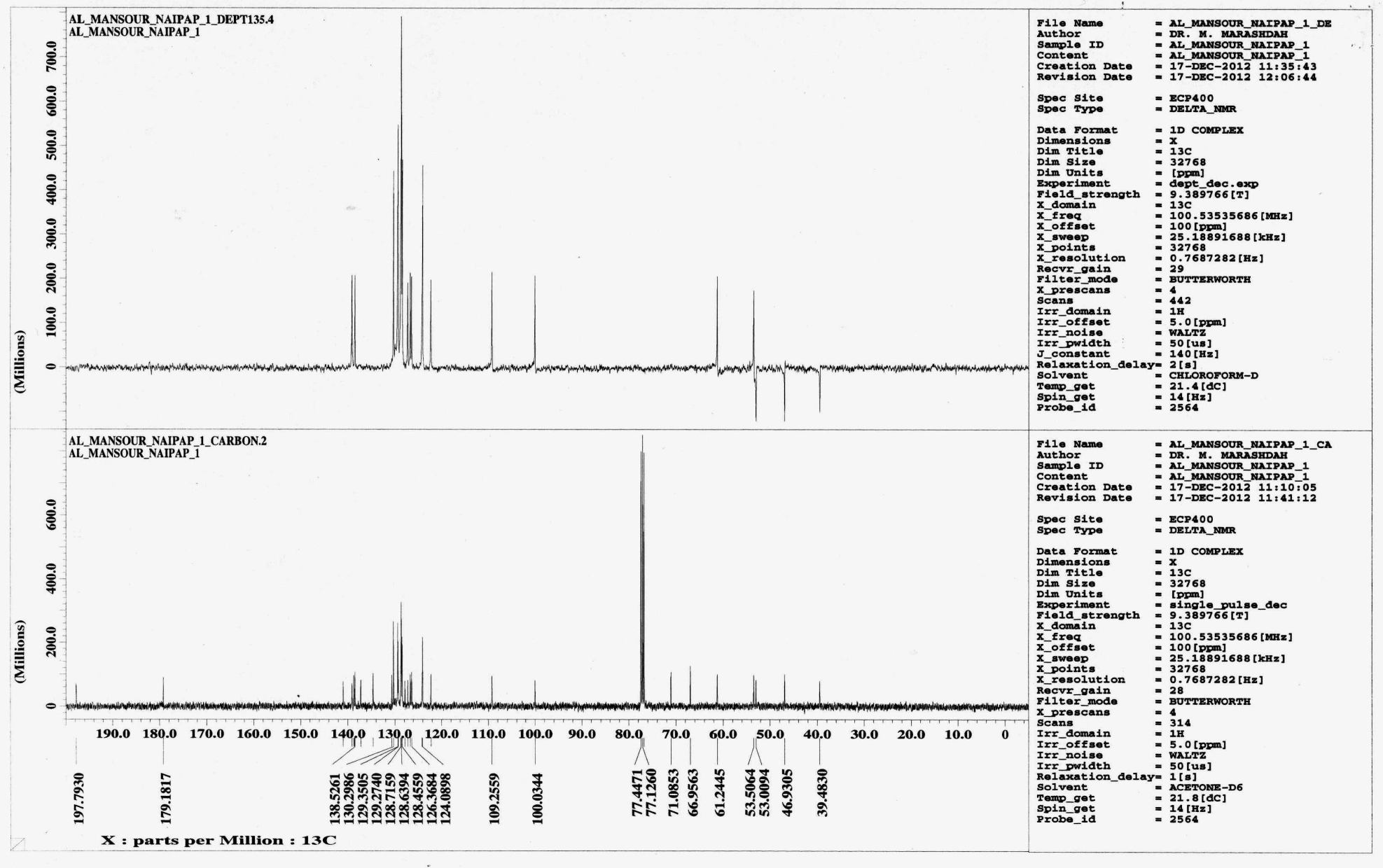 |
| --- |

**Figure S4.** DEPT-135 spectrum of **5a**

| 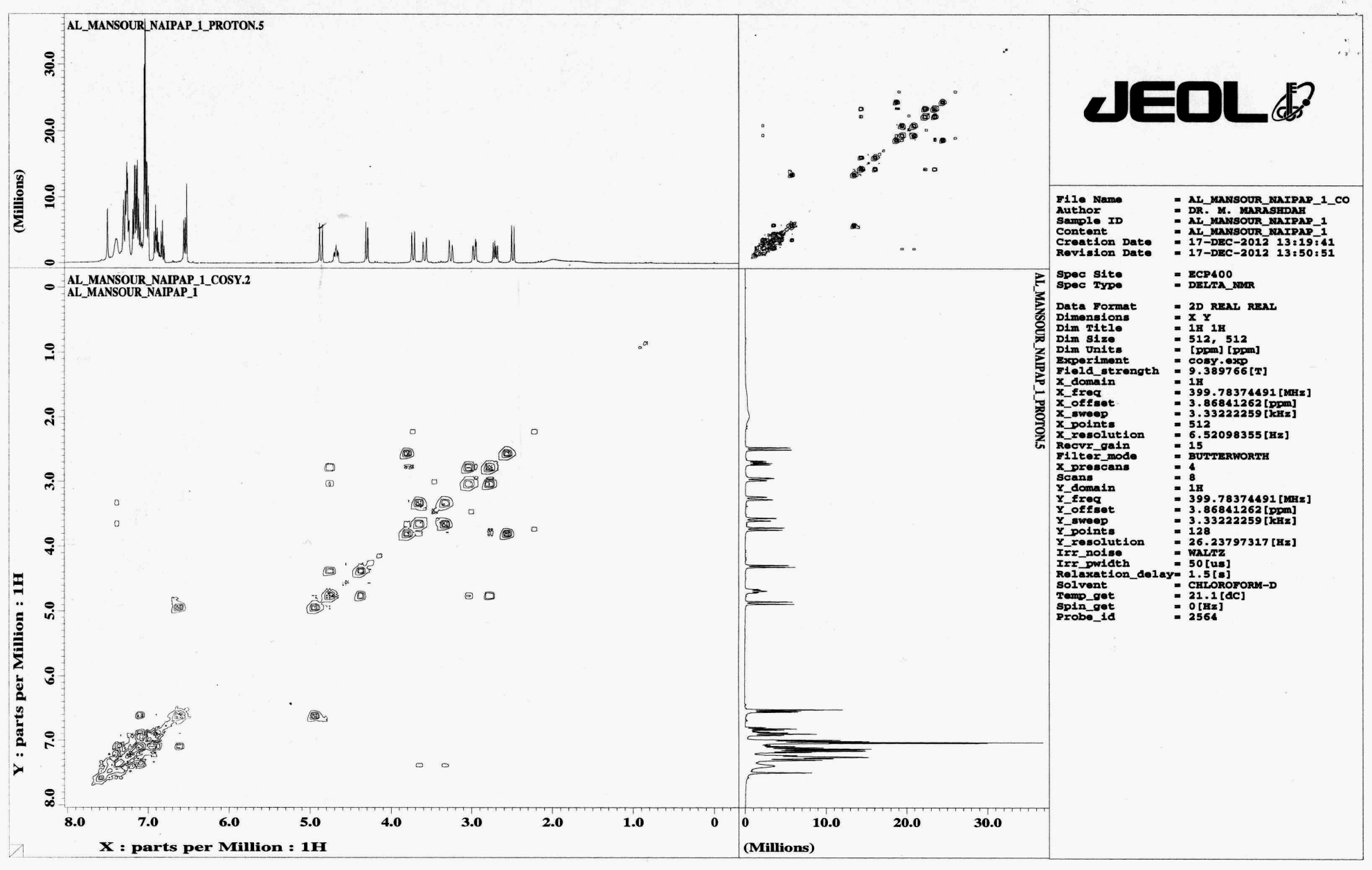 |
| --- |

**Figure S5**. ^1^H, ^1^H-COSY spectrum of 5**a**
